# Modified abdominal packing method in “near miss” patients with postpartum hemorrhages

**DOI:** 10.4274/tjod.79095

**Published:** 2018-09-03

**Authors:** Çetin Kılıççı, Mesut Polat, Mehmet Küçükbaş, Mehmet Baki Şentürk, Resul Karakuş, Çiğdem Yayla Abide, Evrim Bostancı Ergen, İlter Yenidede, Ateş Karateke

**Affiliations:** 1 İstanbul Zeynep Kamil Woman and Children Diseases Training and Research Hospital, Clinic of Obstetrics and Gynecology, İstanbul, Turkey; 2 İstanbul Medeniyet University, Göztepe Training and Research Hospital, Clinic of Obstetrics and Gynecology, İstanbul, Turkey

**Keywords:** Abdominal packing, peripartum hysterectomy, near miss

## Abstract

**Objective:**

To describe a more effective abdominal packing method in patients with disseminated intravascular coagulation following peripartum hysterectomy due to postpartum hemorrhage (PPH).

**Materials and Methods:**

The present retrospective and descriptive study was conducted to document six cases with refractory pelvic bleeding who underwent a second surgery for PPH between January 2016 and December 2017 at İstanbul Zeynep Kamil Woman and Children Diseases Training and Research Hospital.

**Results:**

Karateke packing was performed to control intra-abdominal massive hemorrhages of five women who were referred to our clinic due to PPH who had undergone peripartum hysterectomy and hypogastric artery ligation but hemostasis could not be provided. In addition, a case of hypovolemic shock due to placenta percreta rupture in a woman who had also undergone an emergency hysterectomy and hypogastric artery ligation, which had failed. Hemostasis was provided in all patients. No method-related complication developed.

**Conclusion:**

Karateke packing is a very easy method to perform, it is more effective than the classic abdominal packing technique, with a low complication rate, and most importantly, life-saving in patients undergoing a peripartum hysterectomy due to PPH and thereafter experiencing diffuse hemorrhage.

**PRECIS:** Karateke packing method can be used as the last life-saving method in patients with postpartum hemorrhage.

## Introduction

Postpartum hemorrhage (PPH) is the most common cause of maternal death with high morbidity and mortality rates. The maternal mortality rate has fallen in most countries with advances in reaching blood and blood products, multidisciplinary approaches to cases, and the development of comprehensive protocols. In our country, this rate has fallen from 0.28% to 0.14% in last 10 years^(1,2)^. To maintain sufficient circulation and tissue oxygen supply in PPH, and to stop the hemorrhage simultaneously, interventions such as uterotonic agents, use of Bakri balloon tamponade, B-lynch suture, uterine artery or hypogastric artery ligation, uterine artery embolization, and when necessary, hysterectomy in addition to general obstetric procedures are life-saving^(3,4)^. The incidence of peripartum hysterectomy is 0.2/1000 deliveries in developed countries; however, this rate is 4.43/1000 deliveries in developing countries^(1)^. Despite such efficient treatment options in PPH, repeat laparotomies may be required to control ongoing hemorrhage following a peripartum hysterectomy in patients administered massive transfusion and developing disseminated intravascular coagulopathy (DIC)^(5)^. Abdominal packing is a classic technique that is frequently performed in these “near miss” patients who have no other surgical option. Abdominal packing exerts a mechanical pressure over the low pressure venous and capillary vessels of intra-abdominal deperitonized pelvic surfaces and the surgery region and saves time for transfusion of necessary coagulation factors and blood and blood products such as platelets required for the provision of permanent hemostasis. Additionally, abdominal packing gains time for the clearance of mediators preventing intravascular coagulation by the kidney and liver, and treatment of metabolic problems such as DIC, hypothermia, acidosis, and hypovolemic shock occurring in a patient in the intensive care unit^(6)^. As it was indicated in the review of Touhami et al.,^(7)^ the decision for pelvic packing is generally taken in the event of the development of nonsurgically controllable hemorrhage associated with clinical and laboratory evidence of coagulopathy and not being able to replace blood loss sufficiently despite continuous hemorrhage after an emergency peripartum hysterectomy. Accordingly, the procedure of pelvic packing may be considered as a “rescue ending procedure”.

### Indications for pelvic packing after emergency peripartum hysterectomy

- Hemodynamically unstable patient without optimal resuscitation^(8,9)^

- Stabilization of the patient to enable transfer to an adequate structure^(10)^

- Post hysterectomy hemorrhage with a free interval^(8,11,12)^

- Diffuse vaginal laceration related to pelvic bleeding^(13,14)^

- Presence of extensive hematoma in the absence of the possibility of embolization^(11,14)^

In this study, we aimed to describe a new packing method that we considered as a more effective method in hemorrhage cases requiring a peripartum hysterectomy due to PPH after delivery and in “near miss” patients in whom hemostasis could not be provided despite peripartum hysterectomy and who have no other surgical option and require more than one surgery to control the hemorrhage.

## Materials and Methods

The present retrospective, observational, and descriptive study was conducted to document patients who had a PPH between January 2016 and December 2017 at Zeynep Kamil Research and Training Hospital. The study protocol was approved by the Zeynep Kamil Women and Children Research Hospital local ethics committee (approval number: 120-2015). Informed consent was provided by all participants. A need for intervention had occurred due to a severe PPH in a total of 310 patients. One hundred sixty-four patients (53%) were referred from external centers. Surgical intervention was performed in the external center in 33 patients. The causes of PPH were as follows: postpartum atony in 212 patients, placental invasion anomalies in 81 patients, a uterine rupture in 7 patients, severe retroperitoneal hematoma secondary to lower genital tract injury in 8 patients, and uterine inversion in 2 patients. The causes of PPH and demographic characteristics of patients are shown in Tables 1 and 2. The following interventions were performed: Bakri balloon tamponade in 50 patients, pelvic devascularization and compression suture in 105 patients, local uterine resection in 33 patients, and peripartum hysterectomy in 122 patients.

### Statistical Analysis

A new abdominal packing technique was performed as the final method in six patients undergoing surgery for at least a second time for PPH but not controlled with classic hemostatic methods for whom all surgical methods were exhausted. The data obtained from the study were analyzed using the Statistical Package for the Social Sciences software package (SPSS version 10.0) and the results are expressed as mean ± standard deviation.

## Results

**Figure 1:**
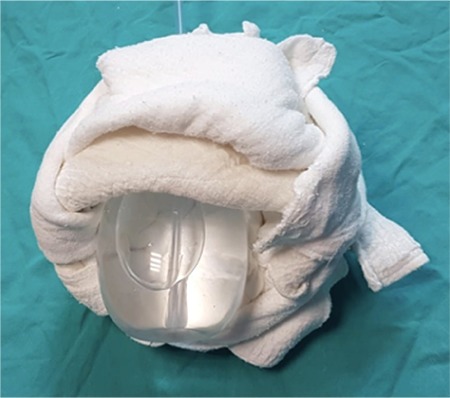
Soaked and squeezed near-hot sponges are wrapped circularly around the balloon

**Figure 2:**
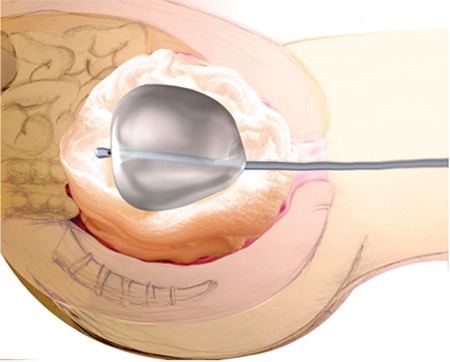
Bakri balloon is inflated with 500-1000 mL saline and put in traction through the vaginal route

**Figure 3:**
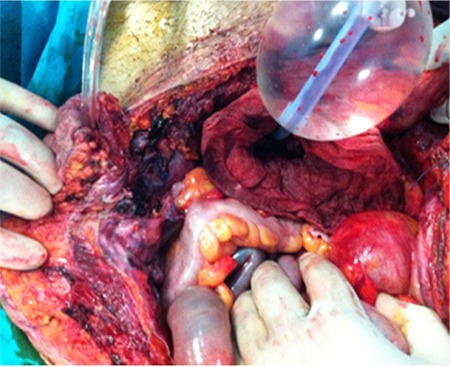
Bleeding stops in vascular structures and hemostasis is provided

The packing technique was performed in five women in whom hemostasis could not be achieved despite a peripartum hysterectomy, and in a patient who presented to the emergency department with the clinical picture of hypovolemic shock due to placenta percreta rupture. Relaparotomy had a severe risk for these 6 “near miss” patients. Therefore, to create a more effective compression considering that sufficient compression could not be provided, a new packing method (Karateke packing) was performed. The packing decision in these patients was made by the obstetric emergency bleeding team of our clinic. The demographic characteristics of these 6 patients are presented in Table 3. The packing technique used by the obstetric bleeding team was performed in the following steps: In Karateke packing, a 1 cm incision is performed in the posterior vaginal wall 1-2 cm from the sutured vaginal cuff, and a Bakri balloon is placed into the abdomen by pulling through the vagina. Six to seven soaked and squeezed near hot sponges are wrapped circularly around the balloon (Figure 1) and then the Bakri balloon is inflated with 500-1000 mL saline and put in traction through the vaginal route (Figure 2). Thus, abdomen of the patient becomes filled with sponges around the inflated Bakri balloon. The balloon exerts compression on the underlying sponges by means of vaginal traction and therefore the pressure is transferred to all surfaces of the surgical region at the same rate and efficiently. In this way, bleeding stops in vascular structures and hemostasis is provided (Figure 3). The distal part of the balloon, under sufficient traction, is fixed to the leg of the patient. Thereafter, the skin is closed without closing the abdominal fascial layers. To prevent Compartment syndrome and perfusion failure of the lower extremities, maintenance perfusion is allowed by releasing the tension on the shaft of the Bakri balloon minimally with 2 hour intervals during the postoperative period. In the event of continuance of bleeding, the balloon is inflated more, more traction is applied, and compression on the underlying sponges is increased. This procedure is continued until hemostasis is provided.

The depacking procedure was performed in all patients after coagulation parameters returned to the normal range and cessation of intra-abdominal hemorrhage following blood and blood products replacement. Preoperative and postoperative laboratory parameters of all our patients are shown in Table 2. In our first patient who received Karateke packing, bilateral hypogastric artery ligation, and B-Lynch suture were performed via laparotomy due to postpartum uterine atony and there was no intra-operative bleeding. A peripartum hysterectomy was performed with a second laparotomy because 2000 mL/h bleeding occurred through the drain of the patient in the intensive care unit postoperatively. Upon continuance of intra-abdominal diffuse bleeding, classic packing was performed perioperatively with 6 pads and the abdomen was closed. At the postoperative 1^st^ hour, the total drained fluid volume was 1500 cc in the drain bag, and a third laparotomy was performed. Karateke packing was performed as the final intervention because there was no surgical option to control the bleeding in the 3^rd^ surgery. In this patient, blood loss of 500 mL/24 h was observed through the drain in the intensive care unit postoperatively.

Peripartum hysterectomy and hypogastric artery ligation were performed in three of our patients due to postpartum atony and peripartum hysterectomy, and bilateral hypogastric artery ligation was performed in one of our patients due to uterine atony and broad ligament hematoma. Upon observation of severe bleeding through the drains of patients followed up in the intensive care unit postoperatively, Karateke packing was performed in a second surgery.

Our last patient was brought to our emergency outpatient clinic directly, she was unconscious with fixed dilated pupils due to placenta percreta rupture at the 34^th^ gestational week of pregnancy. Peripartum hysterectomy and bilateral iliac artery ligation were performed in this patient in an emergency laparotomy. Upon continuance of diffuse bleeding from the peritoneal surfaces and surgical site, Karateke packing was performed. Active bleeding was not observed through the drain in the intensive care unit postoperatively. However, the patient was diagnosed as having cerebrovascular hypoxia at the postoperative 3^rd^ day and died of cardiac arrest on the same day. The clinical characteristics of these patients are presented in Tables 4 and 5.

## Discussion

Pelvic packing techniques can be divided into 2 types: pads or roller gauze (sterile pads bound by suture threads or wrapped in a sterile bag) and balloon pack (Foley catheter or Bakri balloon). There is a difference in physical structure between these 2 types with practical consequences. First, it is easier and faster to assemble and apply the balloon pack because it is ready to be inflated and used immediately, but the pack formed by pads needs to be arranged and connected. Second, it is easier to adjust to match the size of the balloon packs to the size of the hemorrhagic areas with inflation or deflation of the balloon, but the addition or withdrawal of a pad from the constituted pack may be complicated^(11)^. Our packing method is important in regards to being the first to apply two methods in combination, as reported by Touhami et al.^(7)^. The strength of our study is that all of the patients referred to our clinic from external centers comprised “near miss” patients who require massive blood transfusion due to delayed surgical intervention. Classic abdominal packing is not sufficient to stop bleeding. The success of the treatment depends on the success of concurrent medical treatment, intensive care support therapy, and particularly the blood and blood product replacement protocols used. Karateke packing combines the methods defined by Naranjo-Gutiérrez et al.^(10)^ and Charoenkwan K.^(11)^. Karateke packing is more efficient than the classic packing technique and is simpler and more easily applied compared with the method defined by Naranjo-Gutiérrez et al.^(10)^. In a study, packing was performed due to postpartum bleeding associated with uterine atony but hypogastric artery ligation was not performed in one of three patients in whom the Kittipat method was performed^(11)^. At the same time, a complicated clinical picture such as DIC did not accompany this case. Therefore, the efficiency of the method defined by Kittipat in patients developing DIC and requiring massive blood and blood product transfusions such as ours is controversial^(11)^. There is no consensus in the literature regarding the number of sponges required to provide hemostasis related with packing. If the sponges are not squeezed sufficiently, bleeding may continue and packing may result in failure. In the study performed by Deffieux et al.,^(15)^ the authors failed to determine the required number of pads in a successful packing treatment. In Karateke packing, the compression strength exerted on the area of bleeding can be increased with the same number of sponges by increasing the traction force and volume of the intra-abdominal balloon. Thus, mechanical pressure applied to bleeding foci can be increased without having to perform relaparotomy to increase the number of sponges. Deffieux et al.^(15)^ reported the success rate of abdominal packing as 62% in their study. Packing failed in 38% of cases and death occurred in 13^(16)^. In our series, hemostasis was provided with Karateke packing in all 6 patients including a patient in whom bleeding could not be controlled with classic abdominal packing. David Richardson et al.^(16)^ reported that the mortality rate decreased by one-third when abdominal packing was performed in the early period after liver injury. However, the success rate of classic packing would be low in “near miss” patients who are referred in a complicated condition like in our patients. Besides that, Compartment syndrome, which is commonly mentioned in the literature when intra-abdominal pressure exceeds the limit of 20 mm Hg, may develop and this is a serious complication that requires relaparotomy^(17,18)^. In our method, both abdominal fascial layers are not closed and decompression can be provided by releasing the distal tip of the Bakri balloon or by lowering the volume of the balloon when compartment syndrome develops. Symptoms of Compartment syndrome occurred in none of our patients. The depacking procedure is another important problem in abdominal packing. There is no consensus in the literature on this subject. However, it was reported that performing depacking after 24-48 hours reduced the recurrence rate of bleeding. Nicol et al.^(19) ^reported that less bleeding occurred if depacking was performed after 24-48 hours; however, Caruso et al.^(12) ^reported that risk for bleeding increased when sponges were removed earlier than 36 hours. Keeping sponges intra-abdominally for a long time may provide hemostasis but it can cause intra-abdominal adhesions and serious intra-abdominal infection. Abikhaled et al.^(20) ^reported that the rate of abdominal abscess and death was lower when abdominal sponges were kept *in situ* less than 72 hours in 35 women with abdominal injuries. In our patients, the depacking procedure was performed when the bleeding through abdominal drain stopped and DIC laboratory findings regressed (international normalized ratio: <1.2, fibrinogen: >200, hemoglobin: >7). To prevent bleeding from the tissue while removing sponges adhered to visceral organs and surgical site, sponges should be soaked with a hot saline solution, ensured to be separated from the tissue, and removed with slow and gentle movements. The limitation of our method is the need for relaparotomy for the removal of the balloon and sponges.

## Conclusion

Karateke abdominal packing is a very easy method to perform, more effective than the classic abdominal packing technique, with a low complication rate, and most importantly, life-saving in patients undergoing a peripartum hysterectomy due to PPH and thereafter experiencing diffuse hemorrhage.

## Figures and Tables

**Table 1:**
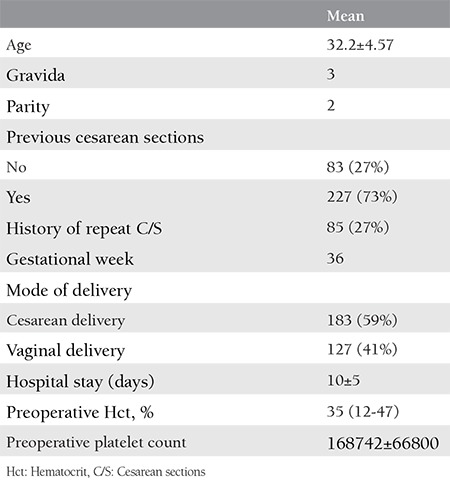
Characteristics of the patients who underwent peripartum hysterectomy because of postpartum hemorrhage (n=310)

**Table 2:**
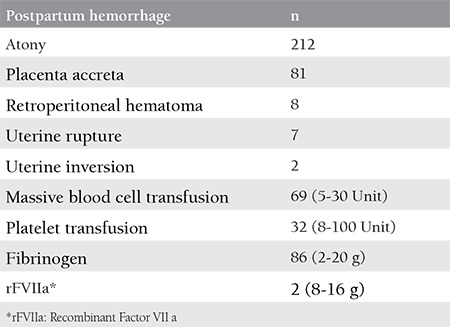
The causes of postpartum hemorrhages and patients requiring transfusion

**Table 3:**
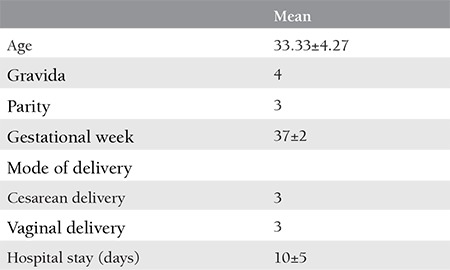
Characteristics of the patients requiring multiple surgeries and abdominal packing following peripartum hysterectomy (n=6)

**Table 4:**
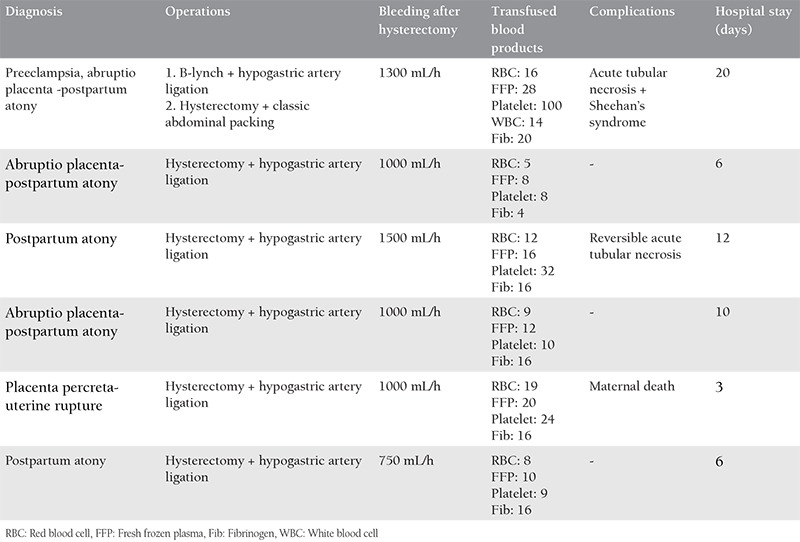
Clinical features of the patients requiring multiple surgeries and abdominal packing following peripartum hysterectomy (n=6)

**Table 5:**
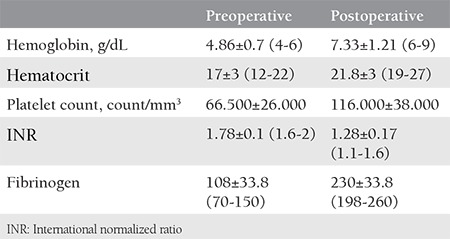
Laboratory values before vs after abdominal packing
